# The association between race and income on risk of mortality in patients with moderate chronic kidney disease

**DOI:** 10.1186/1471-2369-15-136

**Published:** 2014-08-23

**Authors:** Stacey A Fedewa, William M McClellan, Suzanne Judd, Orlando M Gutiérrez, Deidra C Crews

**Affiliations:** 1 Department of Epidemiology, Emory University, Claudia Nance Rollins Building, 3rd Floor, 1518 Clifton Road, NE, Atlanta, GA 30322, USA; 2 Department of Medicine, Emory University, Atlanta, GA, USA; 3 Department of Epidemiology, University of Alabama at Birmingham, Birmingham, AL, USA; 4 Department of Medicine and Division of Nephrology, University of Alabama Birmingham, Birmingham, AL, USA; 5 Division of Nephrology, Department of Medicine, Johns Hopkins University, Baltimore, MD, USA; 6 Welch Center for Prevention, Epidemiology and Clinical Research, Johns Hopkins Medical Institutions, Baltimore, MD, USA

## Abstract

**Background:**

Socioeconomic status (SES) is independently associated with chronic kidney disease (CKD) progression; however, its association with other CKD outcomes is unclear. In particular, the potential differential effect of SES on mortality among blacks and whites is understudied in CKD. We aimed to examine survival among individuals with prevalent CKD by income and race in the Reasons for Geographic and Racial Differences in Stroke (REGARDS) study.

**Methods:**

We examined 2,761 participants with prevalent CKD stage 3 or 4 between 2003 and 2007 in the REGARDS cohort. Participants were followed through March 2013. Mortality from any cause was assessed by income and race (black or white). Low income was defined as an annual household income < $20,000, and was compared to higher incomes (≥$20,000). Cox proportional hazards models adjusted for age, gender, education, insurance, CKD stage, comorbidity and county-level poverty were used to estimate hazard ratios (HR) and 95% confidence intervals (CI).

**Results:**

A total of 750 deaths (27.5%) occurred during the follow-up period. Average follow-up time was 6.6 years among those alive and 3.7 years among those who died. Low income participants had an elevated adjusted hazard of mortality (HR = 1.58, 95% CI 1.24-2.00) compared to higher income participants. Low income was associated with all-cause mortality regardless of race (HR 1.53; 95% CI 1.18-1.99 among blacks and HR 1.38; 95% CI 1.10-1.74 among whites), with no significant statistical interaction between household income and race (p-value = 0.634). However, black participants had a higher adjusted hazard of mortality (HR = 1.30, 95% CI 1.02-1.65) compared to whites, which was independent of income.

**Conclusion:**

Income was associated with increased mortality for both blacks and whites with CKD. Blacks with CKD had higher mortality than whites even after adjusting for important socio-demographic and clinical factors.

## Background

Approximately 14% of adults in the United States have chronic kidney disease (CKD) and have a 60% increased risk of mortality compared to those without CKD [1]. Blacks in the United States have a higher prevalence of advanced CKD and also progress more quickly to end-stage renal disease (ESRD) than whites [2-4]. Several studies have noted a “dialysis survival paradox” where blacks with ESRD undergoing dialysis have better survival compared to whites, despite having generally worse chronic disease outcomes [5-10]. Though the reasons for this paradox are not fully known, it is thought to be in part related to selection advantage of blacks who tend to develop ESRD at younger ages [6]. Some studies suggest lower ESRD mortality among blacks is due to higher mortality rates at earlier stages of CKD; and thus those who survive to progress to ESRD may be more robust [11,12]. Other studies suggest the contrary [6,13-15].

SES is independently associated with CKD prevalence [2] and progression [16]. However, the role of SES on mortality among persons with CKD has received little attention. Furthermore, the potential differential effect of SES on mortality by race/ethnicity is also understudied, though several SES and race interactions have been noted in CKD [4,17-19]. To help clarify the role of SES and its potential differential effect on mortality for blacks and whites, we examined the association between household income and survival by race among participants with CKD in the Reasons for Geographic and Racial Differences in Stroke (REGARDS) study. We hypothesized that lower income would be associated with lower all-cause survival among individuals with CKD, and this association would vary by race.

## Methods

### Study participants and data

The REGARDS study is a population-based national cohort of 30,239 non-institutionalized men and women aged 45 years and older, with almost equal numbers of blacks and whites [20]. Approximately 20% of participants reside in the Stroke Buckle (coastal plains of North Carolina (NC), Georgia (GA) and South Carolina(SC)), 30% reside in the Stroke Belt (remainder of NC, GA, SC, Mississippi, Alabama, Louisiana, Arkansas and Tennessee) and 50% reside in the other 42 contiguous United States.

Briefly, between January 2003 and October 2007, participants completed a telephone interview and in-home examination. Written consent was obtained from each participant. Socio-demographic, household income, and comorbidity data were ascertained through the telephone interview. During an in-home visit, weight and height was collected. Additionally, blood was collected for glucose and serum creatinine measurement. Serum creatinine assays were calibrated to a creatinine standard determined by isotope mass spectrometry [2]. Glomerular filtration rate (GFR) was estimated using the CKD-EPI equation based on a single serum creatinine measurement, which we assumed reflected chronic kidney function [21]. Individuals with an estimated GFR (eGFR) <60 ml/min/1.73 m^2^ were considered to have chronic kidney disease. Individuals with CKD stage 5 at baseline, defined as eGFR <15 ml/min per 1.73 m^2^,were excluded from our analysis. Though participants with prevalent ESRD were excluded at baseline, we examined incident ESRD in addition to mortality as an outcome in our study to account for CKD progression. REGARDS data were linked to the United States Renal Data System (USRDS), which is a national registry of patients with ESRD [22], to identify incident ESRD.

Of the 30,239 REGARDS participants at baseline, 56 participants were excluded for missing data on several key covariates, 25,529 did not have CKD at baseline, and 1,305 were missing data on eGFR. We excluded 167 participants with CKD stage 5 or ESRD at baseline based on REGARDS baseline eGFR and USRDS data. Additionally, 3 participants were excluded due to a death date prior to incident ESRD. Participants with missing household income (n = 445) were excluded leaving 2,761 individuals available for analysis. The REGARDS study was approved by the Institutional Review Boards of the sites involved.

### Outcome

Mortality from any cause was our primary outcome of interest and was assessed through telephone follow-up every 6 months with a proxy that was identified by the participant at baseline [20]. The National Death Index, Social Security Death Index and death certificates were used to identify death events for proxies who could not be found and to confirm the date of death among those reported dead by proxies. We also considered a combined outcome of incident ESRD or mortality. Follow-up data for our study was available through March 2013.

### Primary predictors

Race was self-reported as black or white during the telephone interview. Household income was used as the primary measure of SES and was based on self-reported annual income categories (<$20,000, $20,000-$34,999, $35,000-$75,000 and > $75,000). Low income was defined as < $20,000 based on its proximity to the federal poverty threshold for a family of four ($19,350) in 2005. Medium/high income was defined as ≥ $20,000 [23]. A supplementary analysis using all four income categories was conducted.

### Covariates

Area-based measures including poverty and the Gini Index, were also considered as neighborhood poverty may contribute to kidney disease disparities [24]. County-level Gini Index is a measure of wealth segregation [24,25]. Both Gini Index and county poverty data were based on the 2000 U.S. Census [23]. Education was categorized as no high school diploma, high school diploma, some college and college graduate. Having health insurance was also considered as a covariate. Additional factors included smoking status, grouped as former, current and never smoker. Body mass index (BMI), measured in kg/m^2^, was categorized as underweight (<18.5), normal weight (18.5 to 24.9), overweight (25 to 30) and obese (>30). Stage of CKD, based on National Kidney Foundation Kidney Disease Outcomes Quality Initiative’s staging guidelines; were classified as Stage 3 [eGFRs 30–59 (mL/min/1.73 m^2^)] and Stage 4 [15–29 (mL/min/1.73 m^2^)] [26]. Diabetes was defined based on self-reported medication use, fasting glucose of ≥126 mg/dl or non-fasting glucose ≥ 200 mg/dl. Presence of hypertension was determined by self-reported use of anti-hypertensive medication, systolic blood pressure ≥140 mm Hg or diastolic blood pressure ≥90 mm Hg. Heart disease was based on self-reported history of myocardial infarction, heart attack, or receipt of coronary artery bypass grafting, angioplasty or stenting. Baseline systolic and diastolic blood pressure was measured twice in the left arm with a standard aneroid sphygmomanometer after participants were seated in a chair for three minutes with both feet on the floor. The two blood pressure measurements were averaged [20]. Baseline serum albuminuria (g/dL) was considered as a continuous variable.

### Statistical analysis

Participant characteristics were examined by dichotomized income and race using chi-square and *t*-test statistics (α = 0.05). Two outcomes 1) mortality as well as 2) mortality or incident ESRD was considered. Kaplan Meier curves were used to estimate the proportion of participants who died or developed ESRD over the study period. Log-rank test statistics were used to determine significant differences in survival. Cox proportional hazards models were used to estimate unadjusted and adjusted hazard ratios (HR) and 95% confidence intervals. Adjusted models included household income, race, age, gender, education, geographic location, smoking status, BMI, presence of hypertension, diabetes, heart disease, systolic and diastolic blood pressure, serum albuminuria, county Gini Index and county poverty. The Cox proportional hazards assumption was examined by Schoenfeld residuals. No gross violations in proportionality were detected. Cox models stratified by race were conducted to examine potential differential association of income with mortality. Interaction between race and income was investigated. We also performed sensitivity analyses examining factors related to missing eGFR. All statistical analyses were performed using SAS 9.3 (Cary, NC).

## Results

### Patient characteristics by income and race

The average age of participants was 70.9 years (+/− 9.1), 1,009 (36.5%) participants were black and 745 (27.0%) reported low income. There were a higher proportion of females, non-high school graduates and current smokers in the low income group, and these persons were also slightly older (Table 1). Low income participants had higher prevalence of CKD stage 4, diabetes and hypertension than those of higher income. Black participants were slightly younger (mean age of 69.9 years) compared to whites (mean age 71.5). Furthermore, there were a disproportionate percentage of females and higher prevalence of obesity, CKD stage 4, diabetes, and hypertension among blacks (Table 1).

**Table 1:** **Individual and area level characteristics by individual level income and race, n = 2,761**^**a**^

	**Total N (%)**	**Low income N (%)**	**Medium/high income N (%)**	**P-value**^**b**^	**Black N (%)**	**White N (%)**	**P-value**^**c**^
**Death**	750 (27.54)	493 (24.76)	257 (35.11)	< 0.0001	465 (26.72)	285 (28.99)	
**ESRD**	204 (7.39)	141 (6.99)	63 (8.46)	0.19	65 (3.71)	139 (13.78)	
**Death or ESRD**	873 (31.62)	580 (28.77)	293 (39.33)	< 0.0001	499 (28.48)	374 (37.07)	
**Black Race**	1009 (36.54)	622 (30.85)	387 (51.95)	< 0.0001			
**No Insurance**	100 (3.62)	53 (2.63)	47 (6.32)	< 0.0001	39 (2.23)	61 (6.05)	< 0.0001
**Education**				< 0.0001			< 0.0001
<High School	468 (16.97)	190 (9.44)	278 (37.32)		191 (10.91)	277 (27.48)	
HS Graduate	757 (27.45)	507 (25.19)	250 (33.56)		489 (27.94)	268 (26.59)	
Some College	697 (25.27)	531 (26.38)	166 (22.28)		466 (26.63)	231 (22.92)	
College Graduate	836 (30.31)	785 (39)	51 (6.85)		604 (34.51)	232 (23.02)	
**Age (years)**				0.011			0.0002
<60	329 (11.92)	262 (13)	67 (8.99)		189 (10.79)	140 (13.88)	
60-69	866 (31.37)	642 (31.85)	224 (30.07)		519 (29.62)	347 (34.39)	
70-79	1028 (37.23)	729 (36.16)	299 (40.13)		671 (38.3)	357 (35.38)	
≥80	538 (19.49)	383 (19)	155 (20.81)		373 (21.29)	165 (16.35)	
**Female**	1530 (55.41)	998 (49.5)	532 (71.41)	< 0.0001	890 (50.8)	640 (63.43)	< 0.0001
**Region**				< 0.0001			
Belt	883 (31.98)	598 (29.66)	285 (38.26)		579 (33.05)	304 (30.13)	< 0.0001
Buckle	612 (22.17)	452 (22.42)	160 (21.48)		423 (24.14)	189 (18.73)	
Non-Belt	1266 (45.85)	966 (47.92)	300 (40.27)		750 (42.81)	516 (51.14)	
**CKD Stage 4 **^**d**^	183 (6.63)	109 (5.41)	74 (9.93)	< 0.0001	89 (5.08)	94 (9.32)	< 0.0001
**Smoking Status**				< 0.0001			0.0013
Current	305 (11.07)	191 (9.49)	114 (15.34)		181 (10.34)	124 (12.33)	
Never	1267 (45.97)	921 (45.75)	346 (46.57)		772 (44.11)	495 (49.2)	
Former	1184 (42.96)	901 (44.76)	283 (38.09)		797 (45.54)	387 (38.47)	
**BMI (kg/m**^**2**^**)**				0.0007			< 0.0001
Underweight (<18.5)	32 (1.17)	24 (1.2)	8 (1.09)		24 (1.38)	8 (0.8)	
Normal (18.5-24.9)	601 (21.98)	454 (22.72)	147 (19.97)		436 (25.13)	165 (16.52)	
Overweight (25–29.9)	972 (35.55)	742 (37.14)	230 (31.25)		657 (37.87)	315 (31.53)	
Obese (>30)	1129 (41.29)	778 (38.94)	351 (47.69)		618 (35.62)	511 (51.15)	
**Heart Disease**	848 (31.43)	602 (30.57)	246 (33.74)	0.12	590 (34.32)	258 (26.35)	< 0.0001
**Diabetes**	1023 (37.17)	683 (34)	340 (45.76)	< 0.0001	536 (30.7)	487 (48.41)	< 0.0001
**Hypertension**	2315 (84.27)	1655 (82.58)	660 (88.83)	< 0.0001	1384 (79.4)	931 (92.73)	< 0.0001
**Gini Index**^**e**^				0.003			< 0.0001
<0.442	682 (24.74)	523 (25.98)	159 (21.37)		550 (31.45)	132 (13.1)	
0.442-0.463	674 (24.45)	510 (25.34)	164 (22.04)		469 (26.82)	205 (20.34)	
0.464-0.486	678 (24.59)	480 (23.85)	198 (26.61)		390 (22.3)	288 (28.57)	
>0.486	723 (26.22)	500 (24.84)	223 (29.97)		340 (19.44)	383 (38)	
**County Poverty**				< 0.0001			< 0.0001
<13.41%	692 (25.1)	559 (27.77)	133 (17.88)		532 (30.42)	160 (15.87)	
13.41-16.24%	690 (25.03)	508 (25.24)	182 (24.46)		406 (23.21)	284 (28.17)	
16.24-19.96%	681 (24.7)	490 (24.34)	191 (25.67)		419 (23.96)	262 (25.99)	
>19.96%	694 (25.17)	456 (22.65)	238 (31.99)		392 (22.41)	302 (29.96)	

^a^Participants with missing data items are not included in the percentages. 3 participants were missing data on education, 5 participants were missing data smoking status, 27 participants were missing data on BMI, 63 participants were missing data on heart disease, 9 participants were missing data on diabetes and 14 participants were missing data on hypertension. 4 participants were missing data on area-level poverty and Gini-Index.

^b^Comparing Low Income versus Medium/High Income. Low income was defined as < $20,000 and medium/high income was defined as ≥ $20,000.

^c^Comparing black versus white.

^d^CKD stages 3 and 4 were defined as baseline eGFRs 30–59 (mL/min/1.73 m^2^) and 15–29 (mL/min/1.73 m^2^), respectively.

^e^The Gini Index, a measure of income heterogeneity which ranges from 0 to 1, was considered with 0 indicating perfectly equal income distribution across households in a county and 1 indicating all county income was held within one household.

### Mortality by income level and other SES factors

**Figure 1:**
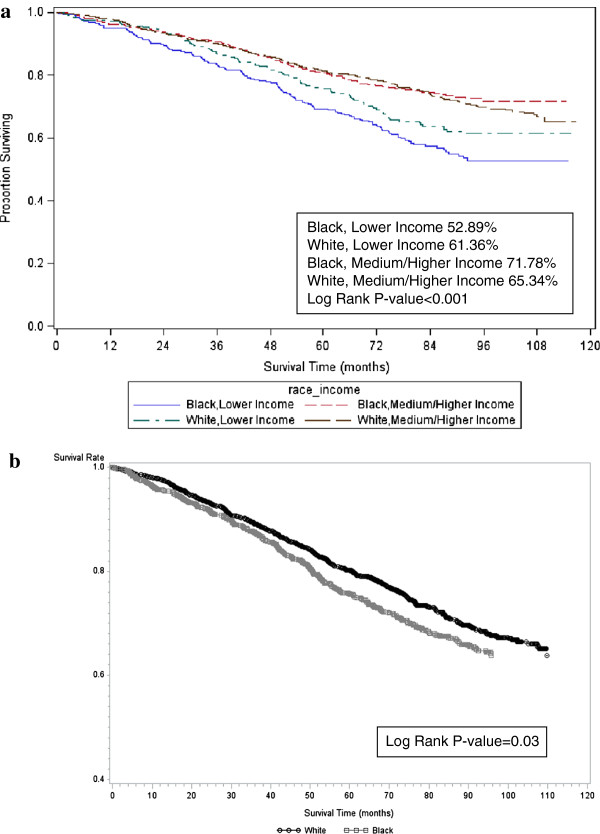
**All cause-mortality among participants with moderate chronic kidney disease in the REGARDS Study. a**. All cause mortality by race and income, n=2,761 **b**. All cause mortality by race, n=2,789.

A total of 750 deaths (27.5%) occurred during follow-up. Average follow-up time was 79.0 months (+/− 24.5) among those living and 44.5 months (+/− 25.0) among those who died. Estimated survival was 52.9% and 71.8% for black participants with low and higher incomes (p-value <0.001), respectively (Figure 1). Estimated survival was 61.4% and 65.3% for white participants with low and higher incomes (p-value <0.001), respectively (Figure 1). Participants with low income had a 58% increased hazard of death in unadjusted analyses (Table 2). In fully adjusted models accounting for demographics, CKD stage, blood pressure, albuminuria, comorbidity and county-level SES, hazard of mortality among low income persons was attenuated, but remained statistically significant (HR = 1.58, 95% CI 1.24-2.00). Hazard ratios for low income were attenuated when ESRD or mortality was considered as the outcome, but remained statistically significant (Table 3). When household income was categorized into four groups participants whose household income was < $20,000 (HR = 1.83, 95% CI 1.31-2.55) had significantly higher adjusted hazards of mortality compared to the highest household income group (>$75,000). However, participants in the medium income groups did not have elevated hazards of mortality compared to the highest income group ($20,000 to $34,000, HR = 1.36, 95% CI 0.99-1.87 and $35,000 to $74, 999, HR = 1.14, 95% CI 0.83-1.58). Residing in counties with 16-20% poverty (HR = 1.40, 95% CI 1.12-1.77) and counties with >20% poverty (HR = 1.33, 95% CI 1.03-1.70) was associated with significantly higher hazards of mortality relative to counties with the lowest proportion of poverty in adjusted models. Gini index, education, and insurance were not significantly associated with mortality in adjusted models.

**Table 2:** Unadjusted and adjusted hazard ratio for mortality and 95% CI among participants with CKD in the REGARDS study, n = 2,761

	**HR (95% CI) model 1 unadjusted**	**HR (95% CI) model 2 adjusted for sociodemographics**^**a**^	**HR (95% CI) model 3 + CKD stage**	**HR (95% CI) model 4 + BMI and smoking**	**HR (95% CI) model 5 + comorbidity**^**b**^	**HR (95% CI) model 6 + area level measures**^**c**^	**HR (95% CI) model 7 + BP and albuminuria**^**d**^
**Income**							
Medium/High	1.00	1.00	1.00	1.00	1.00	1.00	1.00
Low^e^	1.58 (1.36-1.83)	1.72 (1.46-2.03)	1.62 (1.38-1.89)	1.53 (1.30-1.79)	1.46 (1.23-1.74)	1.44 (1.22-1.71)	1.58 (1.24-2.00)
**Race**							
White	1.00	1.00	1.00	1.00	1.00	1.00	1.00
Black	1.17 (1.01-1.35)	1.27 (1.09-1.49)	1.23 (1.06-1.43)	1.24 (1.07-1.45)	1.24 (1.05-1.47)	1.24 (1.04-1.47)	1.30 (1.02-1.65)

^a^Sociodemographic characteristics include age, geographic location, education and gender.

^b^Comorbidities include heart disease, hypertension, and diabetes.

^c^Area Level measures include county poverty and Gini Index. The Gini Index, a measure of income heterogeneity which ranges from 0 to 1, was considered with 0 indicating perfectly equal income distribution across households in a county and 1 indicating all county income was held within one household.

^d^Blood pressure (BP) included systolic and diastolic blood pressure. Serum albuminuria (g/dL) was considered.

^e^Low income was defined as < $20,000 and medium/high income was defined as ≥ $20,000.

**Table 3:** Unadjusted and adjusted hazard ratio for mortality or ESRD and 95% CI among participants with CKD in the REGARDS study, n = 2,761

	**HR (95% CI) model 1 unadjusted**	**HR (95% CI) model 2 adjusted for sociodemographics**^**a**^	**HR (95% CI) model 3 + CKD stage**	**HR (95% CI) model 4 + BMI and smoking**	**HR (95% CI) model 5 + comorbidity**^**b**^	**HR (95% CI) model 6 + area level measures**^**c**^	**HR (95% CI) model 7 + BP and albuminuria**^**d**^
**Income**							
Medium/High	1.00	1.00	1.00	1.00	1.00	1.00	1.00
Low^e^	1.52 (1.32-1.75)	1.62 (1.40-1.88)	1.46 (1.26-1.70)	1.40 (1.20-1.63)	1.29 (1.10-1.50)	1.28 (1.09-1.51)	1.32 (1.06-1.65)
**Race**							
White	1.00	1.00	1.00	1.00	1.00	1.00	1.00
Black	1.45 (1.27-1.66)	1.57 (1.37-1.80)	1.52 (1.32-1.75)	1.55 (1.35-1.79)	1.52 (1.31-1.77)	1.52 (1.30-1.78)	1.63 (1.31-2.01)

^a^Sociodemographic characteristics include age, geographic location, education and gender.

^b^Comorbidities include heart disease, hypertension, and diabetes.

^c^Area Level measures include county poverty and Gini Index. The Gini Index, a measure of income heterogeneity which ranges from 0 to 1, was considered with 0 indicating perfectly equal income distribution across households in a county and 1 indicating all county income was held within one household.

^d^Blood pressure (BP) included systolic and diastolic blood pressure. Serum albuminuria (g/dL) was considered.

^e^Low income was defined as < $20,000 and medium/high income was defined as ≥ $20,000.

### Mortality by race

Unadjusted survival for blacks was higher than whites among those with medium or high income; however, the opposite was true for low income where blacks had worse survival as shown in Figure 1. However, a test for interaction for race and income was not statistically significant (p = 0.149). Black participants had a higher, but not statistically significant, unadjusted hazard of mortality compared to whites (HR = 1.15, 95% CI 0.99-1.33). The fully adjusted HR for blacks was significantly higher compared to whites (HR = 1.30, 95% CI 1.02-1.65) (Table 3). When ESRD and mortality were considered as a composite outcome, there was a strong association between black race and ESRD or mortality. In unadjusted analyses black participants had a 1.45 (95% CI 1.27-1.66) hazard of ESRD or mortality compared to whites (Table 3) and this remained significant following adjustment for other factors [HR 1.63 (95% CI 1.31-2.01)].

### Mortality by income level stratified by race

Low income was associated with an 82% increase in HR for mortality for blacks and a 38% increase for whites in unadjusted models (Table 4). In adjusted models, low income corresponded with a 59% and 34% increase in HR for blacks and whites, respectively (Table 4). However, there was no significant statistical interaction between race and low income status (p-value = 0.448). When the composite outcome of ESRD or mortality was considered, hazard ratios for low income were dampened for both blacks and whites. Among whites, the unadjusted HR for low income was 1.33 (95% CI 1.08-1.63) and the fully adjusted HR was no longer statistically significant (HR = 1.23, 95% CI 0.88-1.70). For blacks, the unadjusted HR associated with low income and incident ESRD or mortality was 1.55 (95% CI 1.27-1.90) and the fully adjusted HR was 1.59 (HR = 1.59, 95% CI 1.16-2.18). There was no significant interaction between race and income on mortality (p-value = 0.783).

**Table 4:** Adjusted hazard ratios and 95% confidence intervals for mortality or ESRD associated with low income by race/ethnicity among participants with CKD in the REGARDS study, n = 2,761

**Model number**	**Variables included**	**Black HR (95% CI)**	**White HR (95% CI)**	**P-value for interaction of race and income**
1	Low income	1.82 (1.44-2.30)	1.38 (1.12-1.70)	0.075
2	+age	1.69 (1.34-2.14)	1.27 (1.03-1.60)	0.095
3	+gender	1.98 (1.55-2.52)	1.55 (1.24-1.93)	0.124
4	+education	2.04 (1.57-2.65)	1.51 (1.20-1.89)	0.135
4	+geographic location^a^	2.03 (1.56-2.64)	1.51 (1.21-1.90)	0.161
5	+CKD stage	1.94 (1.49-2.53)	1.46 (1.16-1.83)	0.236
7	+smoking, BMI and comorbidity^b^	1.62 (1.24-2.13)	1.42 (1.14-1.77)	0.062
8	+county Gini Index score and poverty	1.59 (1.21-2.08)	1.34 (1.06-1.70)	0.448

^a^Geographic location includes: stroke belt versus non-stroke belt.

^b^Comorbidities include heart disease, hypertension, and diabetes.

### Mortality by other patient characteristics

Several factors were independently associated with an increased adjusted hazard of mortality including age, CKD severity (stage 4 versus stage 3 HR = 2.26, 95% CI 1.80-2.84), current smoking (HR = 2.58, 95% CI 2.02-3.27) and former smoking (HR = 1.34, 95% CI 1.14-1.59), as compared to never smoking. The presence of diabetes (HR = 1.52, 95% CI 1.30-1.79) or heart disease (HR = 1.59, 95% CI 1.36-1.85) was also associated with increased hazard of mortality in fully adjusted models. Similar associations were observed when incident ESRD or mortality were considered as an outcome.

### Sensitivity analysis

We compared participants with missing eGFR (n = 1,305) with participants with non-missing eGFR in our study (2,761). The baseline prevalence of heart disease, diabetes, and hypertension was 18.4%, 27.7%, and 67.8%, respectively. Among those with missing eGFR mortality by the end of follow-up was lower among those missing eGFR (19.5%). In a multivariable model examining demographic factors related to missing eGFR, black race (OR = 1.73, 95% CI 1.48-2.01) and gender (OR = 1.18, 95% CI 1.01-1.38) were both positively associated with missing eGFR. Income was not associated with missing eGFR.

## Discussion and conclusions

Among black and white adults with moderate CKD, low income was associated with greater mortality compared to medium/higher income. This association was partially explained by differences in demographic characteristics, CKD stage and comorbidity; however, even after adjustment for these factors, the relation persisted. Those with medium income did have an increased hazard of mortality compared to participants with higher income. Lower income black and white participants had higher mortality than higher income persons, and the consideration of the combined outcome of incident ESRD or mortality did not alter these findings.

Increased mortality among low income participants in our study is likely multi-factorial. Plantinga et al. reported significantly higher disability among lower income individuals with CKD, which may also be related to inadequate treatment and self-management [27]. Comorbid conditions, including diabetes and hypertension, are also less likely to be properly managed among those with lower socioeconomic status. For example, in a study examining blood pressure control among CKD stage 3 and 4 patients, 55% of those in the lowest income category had uncontrolled blood pressure compared to 44% of those in the highest [28]. In addition to the role of treatment and management of CKD, poverty may impact mortality through other, more direct, pathways including stress and inflammation [29,30]. These factors have been shown to be important predictors of all-cause mortality [30,31].

In fully adjusted models, we found low income black and white participants had similar risk of mortality, suggesting the impact of household income on mortality is similar for both race groups. Previous studies have noted a particularly detrimental effect of low income on blacks with regards to CKD prevalence and severity. Lower income was associated with increased odds of CKD among blacks but not whites in an urban population [18] and lower income was associated with higher albuminuria in a study of REGARDS participants [4]. In line with a previous study noting higher mortality among blacks with pre-dialysis CKD [11], we found the same after adjustment for important confounders. Mehrotra et al. found a 78% increased risk of mortality among black CKD persons >65 years of age using a random-sample of National Health Interview Survey Data. In contrast, Newsome et al. noted a survival advantage for blacks with more advanced CKD (eGFR <44 ml/min per 1.73 m^2^) and reported a slight survival advantage for whites among participants with less severe CKD (eGFR 45–60) when they examined Medicare patients admitted for acute myocardial infarction. Similarly, Kovesdy et al. found a survival advantage among blacks as CKD stage progressed among males in the U.S. Veterans Administration (VA) health system [6]. Unlike these studies, we observed a survival advantage for whites compared to blacks with moderate CKD. These discordant findings could be due to differences in study populations. The aforementioned studies were based on inpatient Medicare and male VA patients, respectively, whereas the REGARDS cohort is well-characterized population-based sample, which is a strength of this study.

In addition to higher mortality among black participants in our study, progression to ESRD was also higher among blacks. These findings are consistent with previous studies indicating faster progression from CKD to ESRD among blacks compared to whites [32]. CKD can seldom be reversed; however its progression can be mitigated and controlled through blood pressure management, physical activity, dietary interventions and medication [33]. Delivery of such interventions may be less than adequate among black patients with CKD.

Our study had several limitations. Although we accounted for disease severity at baseline, we were unable to assess CKD disease management, which may partially account for survival variations by income level. Furthermore, we excluded 1,305 participants with missing eGFR measurements. A sensitivity analysis revealed that participants missing eGFR had higher survival and lower comorbidity burden than participants included in our study, but were not significantly different in terms of age. These observations provide some evidence that those missing eGFR, on average, may not have had chronic kidney disease at baseline and were therefore not eligible to be in our study. An additional limitation of our study was the use of a single eGFR measurement to define CKD, which is subject to measurement error and misclassification and preventing an assessment of disease progression. Therefore, we can only speculate on mechanisms through which low income and black race impact mortality. Future longitudinal investigations of SES, race and mortality among persons with CKD are encouraged to help further elucidate these relationships. We also used the same eGFR cutpoints to define CKD for both blacks and whites. However, while, it has been suggested that different cutpoints should be used to define CKD for these groups, a recent study supports the use of the current CKD definition and staging for both blacks and whites [34]. We relied on self-reported household income, which has not been validated and is likely to be misclassified as participants may overestimate their income. However, misclassification of income may have been mitigated by having participants select their income from four categories in the telephone interview compared to an open-ended question. Additionally, we did not have information on household size. The average household size in the US was approximately 2.6 persons in 2010, which varied greatly among younger age groups [35]. Given the relatively older age in our study there may be limited variability in household size. Although several measures of SES were included in our analyses, we did not have information on utilization of safety net programs or community health centers among the uninsured which has been shown to be associated with higher quality of care for chronic diseases [36].

The limitations of our study are balanced by it being one of the first to examine the relation of income, race and mortality in a population-based study of men and women with CKD. We have shown that income is an important predictor of mortality for both blacks and whites with CKD and blacks have higher mortality even after adjusting for important socio-demographic and clinical factors. The reasons for such disparities are likely multifactorial and future longitudinal investigations of SES, race and mortality among persons with CKD are warranted.

## Competing interests

Additional funding was provided by an investigator-initiated grant-in-aid from Amgen Corporation. Amgen did not have any role in the design and conduct of the study, the collection, management, analysis, and interpretation of the data, or the preparation or approval of the manuscript. The manuscript was sent to Amgen for review prior to submission for publication.

Ms. Fedewa is supported by Emory University’s Laney Graduate School and Department of Epidemiology. Dr. Crews was supported by the Harold Amos Medical Faculty Development Program of the Robert Wood Johnson Foundation, Princeton, NJ and grant 1K23DK097184-01 from the National Institute of Diabetes and Digestive and Kidney Diseases (NIDDK), Bethesda, MD.

## Authors’ contributions

DC, WM, and SF conceived of the study, and participated in its design of the study. All authors helped to draft the manuscript. SF performed the statistical analyses. All authors read and approved the final manuscript.

## Pre-publication history

The pre-publication history for this paper can be accessed here:

http://www.biomedcentral.com/1471-2369/15/136/prepub
